# Interstitial Lung Disease in Anti-Synthetase Syndrome

**DOI:** 10.31138/mjr.30.3.186

**Published:** 2019-09-30

**Authors:** Evangelia Kourkouni, Georgios Mitsogiannis, Theodora Simopoulou, Christos Liaskos, Christina G. Katsiari, Zoi Daniil, Konstantinos Gourgoulianis, Dimitrios P. Bogdanos, Lazaros I. Sakkas

**Affiliations:** 1 Department of Rheumatology and Clinical Immunology;; 2 Department of Respiratory Medicine, University General Hospital of Larissa, Faculty of Medicine, University of Thessaly, Larissa, Greece

**Keywords:** anti-synthetase syndrome, anti-Jo1, interstitial lung disease, Raynaud’s phenomenon

## Abstract

Anti-synthetase syndrome is an autoimmune disorder characterized by the presence of autoantibodies against aminoacyl transfer RNA (tRNA) synthetases, and myositis, interstitial lung disease (ILD), arthritis, fever and Raynaud’s phenomenon (RP). We present a 54-year-old woman, who complained of fatigue, low-grade fever, myalgias, arthralgias, RP and dyspnoea on exertion. Chest CT scan revealed features of interstitial lung disease. Due to rapid deterioration of her lung function, she required oxygen support. The patient did not respond to empiric treatment with antibiotics. Autoantibody testing was remarkable for ANA positivity (1/160) and high-titre anti-Jo1 positivity. A diagnosis of anti-synthetase syndrome was made and the patient was placed on high-dose corticosteroids and rituximab with significant improvement. At 1-year follow up, she remains in good condition, without the need for oxygen supplementation.

## INTRODUCTION

Anti-synthetase syndrome (aSS) is a rare autoimmune disorder characterized by the presence of antibodies against aminoacyl-transfer RNA synthetase (ARS) and a variable constellation of manifestations; most commonly interstitial lung disease (ILD), myositis, and arthritis. These manifestations occur in 60–95% of cases, whereas other, less common manifestations occurring in ∼40% of cases include Raynaud’s phenomenon (RP), fever and mechanic’s hands.^1^ By far the most common ARS antibody is anti-Jo1, which targets histidyl-tRNA synthetase (histidyl-RS) and occurs in 73% of aSS patients.^2^ Other less common ARS antibodies are against PL-12 (alanyl-RS) (in 14% of patients), PL-7 (threonyl-RS) (in 9% of patients), OJ (isoleucyl-RS), and EJ (glycyl-RS), KS (asparaginyl-RS), Zo (phenylalanyl-RS), and YRS/Tyr (tyrosyl-RS).^3^ Patients with anti-synthetase syndrome often suffer from severe and rapidly progressive ILD. In fact, life expectancy is curtailed in aSS and the major cause of death is ILD and pulmonary hypertension. The 5-year survival is 90% in anti-Jo-1-positive patients and 75% in non-Jo-1-positive patients but the 5-year survival can be as low as 31%.^4^ Thus, it is important to recognize the syndrome early and promptly institute immunosuppressive treatment. Herein, we describe a patient with anti-synthetase syndrome with rapidly progressive and severe ILD.

## CASE PRESENTATION

**Figure 1.**
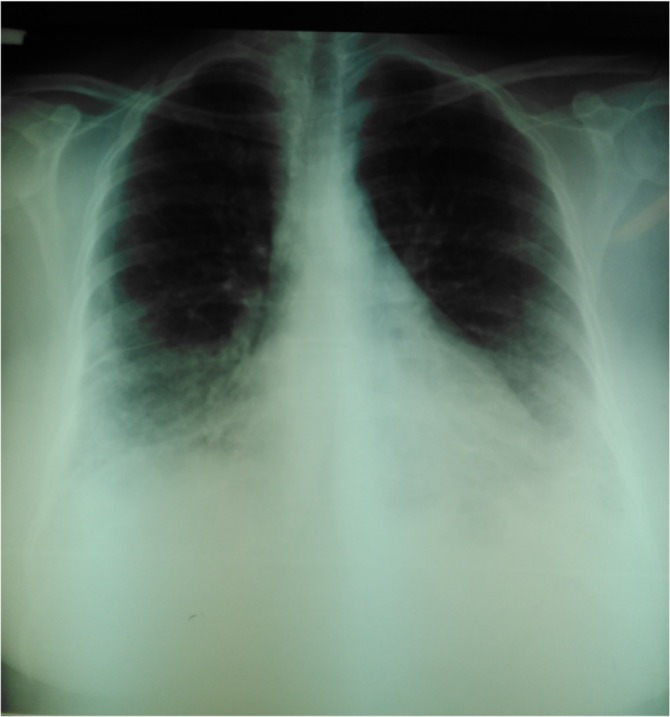
Chest x-rays showing increased markings in lower lung fields.

**Figure 2.**
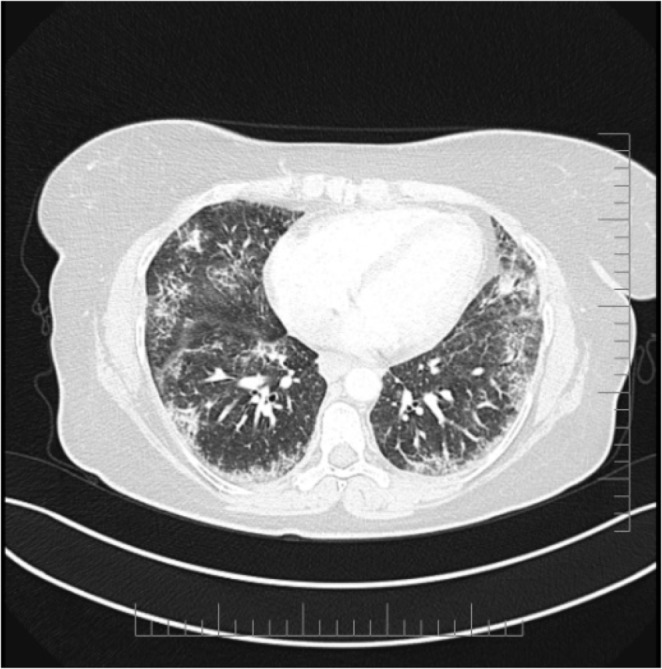
Chest CT scan showing thickened linear markings, ground glass opacities, and mild bronchiectasis.

A 54-year-old Caucasian woman presented to a private physician with a twenty days history of fatigue, low grade fever, myalgias, arthralgias, Raynaud’s phenomenon and dyspnoea on exertion. She was initially treated with 20mg of methylprednisolone as undifferentiated arthritis, and symptoms were improved. Attempts to taper down steroids led to relapse of manifestations. A new chest X-ray revealed increased lineal markings in lower lung fields (*Figure 1*) and a chest CT scan showed linear markings, ground glass opacities and mild bronchiectasis (*Figure 2*). Laboratory tests were remarkable for increased inflammation indices (CRP 3.5mg/dL [normal, <0.5 mg/dL], ESR 42 mm]), mild creatinine kinase elevation (CPK 185 U/L, normal, <145) and a positive ANA test (1/160, normal <1:80). Methylprednisolone was discontinued and empiric antibiotics (clarithromycin, moxifloxacin), antiviral (oseltamivir) and bronchodilators were prescribed, for presumed respiratory infection, without benefit.

She was referred to the Emergency department of our Hospital and admitted to the Department of Rheumatology and Clinical Immunology for further evaluation and treatment.

On admission, she had mild hypoxia with reduced arterial oxygen saturation (SaO_2_ saturation, 90%–92%, pO_2_ 65 mmHg on FiO_2_ 21%), and arterial blood pressure was normal. The patient had low-grade fever up to 38° C (two spikes per day). Inflammation markers were elevated (CRP 2.2 mg/dL, ESR 23mm). The rest of the basic laboratory work-up is shown in *Table 1*. Pulmonary function tests revealed mildly restricted pattern with forced vital capacity (FVC) 74%, forced expiratory volume in 1s (FEV1) 73%, and FEV1/FVC 100%.

**Table 1.** Laboratory tests during hospital stay.

**Test**	**At admission**	**At exit**
Haematocrit (%)	40	39
WBC (×10^3^/μL)	8800	14300
PLT (×10^3^/μL)	367	364
CRP (mg/dL, normal <0,5)	2,2	0,7
ESR (mm)	35	23
Lactate dehydrogenase (IU/L, normal <245)	377	273
Creatine phosphokinase (U/L, normal <145)	241	88

Differential diagnosis of diffuse pulmonary infiltrates, fever, hypoxia and elevated inflammatory markers included respiratory infections, and autoimmune pulmonary/rheumatic diseases. The latter included hypersensitivity pneumonitis, sarcoidosis, systemic lupus erythematosus, Sjögren syndrome, systemic sclerosis, rheumatoid arthritis, vasculitis, and myositis. An autoantibody panel with RF, anti-CCP, ANCA, and ANA was ordered.

**Figure 3.**
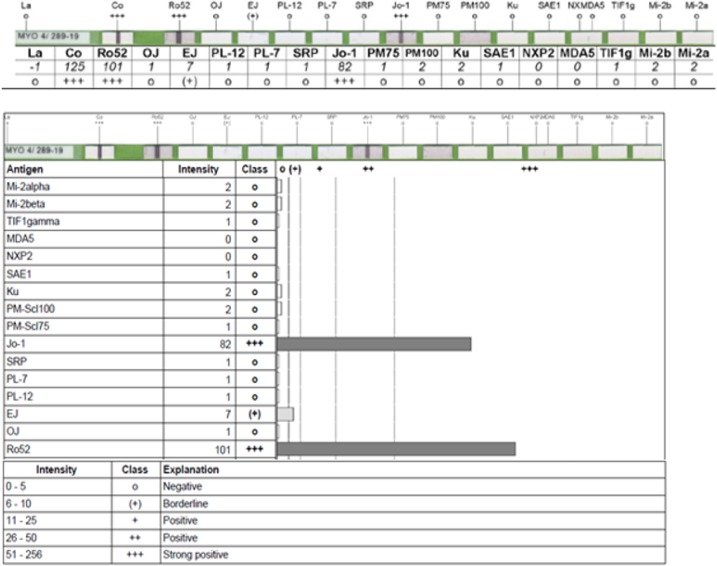
Line immunoblot assay showing positivity for Jo-1 and Ro52 autoantibodies.

Legionella and Pneumococcal antigen urinary tests, serological tests for EBV, CMV, HIV, Parvo B19, myco-plasma and brucellosis, and Mantoux skin test were negative. Blood and urine cultures were sterile. The patient could not provide sputum for culture or immunofluorescence for *Pneumocystis jirovecii*. Beta D-glucan test for fungal infection was not available as a routine test in the microbiology laboratory of the hospital. The patient was empirically treated with broad spectrum antibiotics (intravenous piperacillin-tazobactam) without response. She progressively deteriorated with dyspnoea, tachypnoea, low pO_2_ (49 mmHg) requiring nasal oxygen supplementation, and rising CRP (6.3 mg/dl). Pulmonary embolism and acute heart failure were ruled out. *Pneumocystis jirovecii* pneumonia could not be excluded, and empirical treatment with co-trimoxazole (15 mg/kg/day) plus prednisone according to the treatment guidelines was initiated. Initially, the patient’s clinical status and laboratory findings (CRP 0.7 mg/dl) were improved, but with prednisolone tapering, fever reappeared, and oxygen supplementation was again needed. Therefore, the possibility of an ILD associated with immune-mediated rheumatic diseases was very likely. Systemic sclerosis, systemic lupus erythematosus, Sjögren’s syndrome, and vasculitis were unlikely, since there was no other feature of these diseases. Rheumatoid arthritis was still a possibility, but anti-synthetase syndrome became the most likely diagnosis. In our department, we perform an autoantibody profile for myositis-anti-synthetase syndrome by line immunoassay (*Figure 3*). This multiplex test revealed very strong positivity for anti-Jo-1 (82 Arbitrary units, AU, cut off, 10) and anti-Ro52 antibodies (101 AU, cut off, 10). Antibodies against Mi-2 alpha, Mi-2 beta, TIF1γ, MDA5, NXP2, SAE1, Ku, PM-Scl100, PM-Scl75, SRP, PL-7, PL-12, EJ, and OJ) were all negative. Serum creatine phosphokinase (CPK) levels and electromyography were normal. Therefore, a diagnosis of anti-synthetase syndrome was made on the basis of ILD, fever, arthralgias, RP, and anti-Jo1 autoantibodies. High dose prednisolone was initiated along rituximab (1g on day 0 and day 15). The patient exhibited a quick improvement which continued during the following months with tapering of corticosteroids. At 1-year follow up, the patient is on 5 mg of prednisolone per day and rituximab (day 0–day 15) every six months. She is in good health, without the need of oxygen, and has started her work again. Chest x-rays and pulmonary function tests were normal.

## DISCUSSION

Our case highlights the need for early recognition and treatment of aSS-ILD. Although aSS is associated with myositis, a patient may initially present with no myositis.^1^ On the other hand, ILD is the most prevalent manifestation of aSS occurring in 86–95% of patients.^5,6^ ILD frequently dominates the clinical picture at presentation and may rapidly progress to pulmonary failure. In fact, aSS-ILD is more prevalent and severe rather than myositis-associated ILD.^4^ ILD is more severe in anti-PL12/PL7 patients rather than anti-Jo-1 patients.^4^ In fact, patients with anti-PL12/PL7 antibodies have predominant ILD, often without muscle involvement, whereas patients with anti-Jo-1 antibodies have more diffuse phenotype.^2,5^ The presence of anti-Ro52 antibody in patients with aSS may be associated with rapidly progressive ILD^6,7^ and cancer.^7^ It should be noted that patients with ILD should also be tested for associated anti-signal recognition particle antibodies, including anti-MDA-5 (RNA helicase) and anti-Mi-2 (nuclear helicase). CT findings of aSS-ILD mostly include areas of ground glass opacities and reticulation, predominantly in lower lung lobes, and peribronchial lesions. Less frequently (∼35%) there is organizing pneumonia with fibrosis.^8^ Patients with aSS may also have myocarditis and pulmonary hypertension.^4^ Myocarditis occurs in 34% of aSS patients and may be asymptomatic or present with acute/subacute heart failure.^9^

No randomized control trials have been conducted in aSS, current therapy is off-label and based on case series and case reports, and includes corticosteroids along with an immunosuppressant, such as azathioprine, mycophenolate mofetil, calcineurin inhibitors (tacrolimus), rituximab and cyclophosphamide.^4^ Rituximab seems to be a very effective option with marked improvement in FVC in patients with connective tissue disease and ILD, who have anti-Jo1 antibodies.^10^ Significant improvement after treatment with rituximab is noted more frequently in anti-Jo1 antibody-positive compared to anti-Jo1 antibody-negative patients with inflammatory myopathies. In our case, an improvement in CT imaging features, as well as at FVC and DLCO tests, was observed over time. It must be noted that the greatest benefit was seen at the 3-year follow-up.

In conclusion, early recognition of aSS and investigation for major organ involvement is required for the optimal outcome of patients with this syndrome.
